# Assessing the relationship between contact sensitization and eczema in adolescents and adults on the example of nickel and neomycin: preliminary results of a meta-analysis of epidemiological data

**DOI:** 10.1186/2045-7022-5-S1-O20

**Published:** 2015-03-11

**Authors:** Danuta Plichta, Radoslaw Spiewak

**Affiliations:** 1 Jagiellonian University Medical College, Dept of Experimental Dermatology and Cosmetology, Krakow, Poland

## Background

Throughout literature, positive patch tests to nickel were found in 8.0–51.5% patients with eczema and in 5.9–27.8% general population, the respective figures for neomycin being 0.0–40.0% and 0.0–2.9%, respectively. As these ranges for eczema patients and general population overlap, it is not clear how strong is the association between the disease and contact sensitization to respective haptens. The aim of the study was to analyze the putative relationship between contact sensitization to nickel or neomycin and eczema with the means of meta-analysis of available data for adolescents and adults.

## Method

A systematic review of published data on the prevalence of sensitization to nickel and neomycin in adolescent and adult eczema patients and the general population. Patch test data from studies of respective populations that were comparable with regard to geographical location, year of study, demographics of the study group and patch test protocols were extracted and pooled to calculate estimated odds ratios (eOD) and estimated attributable risk ratios (eAR).

## Results

Data from patch test studies carried out in Denmark, Germany, Italy, North America and Poland have met the inclusion criteria. In case of nickel, the eOR calculated based upon these data varied greatly from 0.8 in Denmark (eAR= –1,6%; weak protective effect) to eOR=46.8 in Italy (eAR=50.4%; very strong relationship between nickel sensitization and eczema). For neomycin, the results seemed more consistent with regard to the direction of the relationship, with eOR ranging from 2.5 (eAR=2.1%) in Germany to infinity (sensitization to neomycin observed only among people with eczema, eAR=3.2%) in Poland. The major obstacle in constructing the eOR and eAR models consists of the scarcity of patch tests results for the general population, and the difficulty of matching the few available studies with data from clinical studies that would fit with regard to the geographical location, year of performing the studies, demographics of the group, and patch test protocol.

## Conclusion

We believe that such meta-analyses of epidemiological data may be a useful tool while looking at the clinical relevance of patch tests in individual patients.

